# A retrospective evaluation of the impact of a dedicated obstetric and neonatal transport service on transport times within an urban setting

**DOI:** 10.1186/1865-1380-4-28

**Published:** 2011-06-14

**Authors:** Shaheem De Vries, Lee A Wallis, David Maritz

**Affiliations:** 1 Division of Emergency Medicine, University of Cape Town and Stellenbosch University, Cape Town, South Africa

## Abstract

**Objective:**

To determine whether the establishment of a dedicated obstetric and neonatal flying squad resulted in improved performance within the setting of a major metropolitan area.

**Design and Setting:**

The Cape Town metropolitan service of the Emergency Medical Services was selected for a retrospective review of the transit times for the newly implemented Flying Squad programme. Data were imported from the Computer Aided Dispatch programme. Dispatch, Response, Mean Transit and Total Pre-hospital times relating to the obstetric and neonatal incidents was analysed for 2005 and 2008.

**Results:**

There was a significant improvement between 2005 and 2008 in all incidents evaluated. Flying Squad dispatch performance improved from 11.7% to 46.6% of all incidents dispatched within 4 min (*p *< 0.0001). Response time performance at the 15-min threshold did not demonstrate a statistically significant improvement (*p *= 0.4), although the improvement in the 30-min performance category was statistically significant in both maternity and neonatal incidents. Maternity incidents displayed the greatest improvement with the 30-min performance increasing from 30.3% to 72.9%. The analysis of the mean transit times demonstrated that neonatal transfers displayed the longest status time in all but one of the categories. Even so, the introduction of the Flying Squad programme resulted in a reduction in a total pre-hospital time from 177 to 128 min.

**Conclusion:**

The introduction of the Flying Squad programme has resulted in significant improvement in the transit times of both neonatal and obstetric patients. In spite of the severe resource constraints facing developing nations, the model employed offers significant gains.

## Introduction

Maternal and child health is one of the main focusses of the World Health Organisation's Millennium Development Goals. These have formed the basis of national strategic initiatives of both Government and Non-Governmental Organisations [1]. In this regard, many of the initiatives in the South African health context have largely focussed on hospitals or primary health care, however, very little attention has focussed on the impact of Emergency Medical Services (EMS) achieving these goals.

METRO EMS is a state run ambulance service providing essential pre-hospital emergency care to the population of the Western Cape of South Africa. It serves a total area of 129,526 km^2 ^with an estimated total population of 5,400,000 people [2]. METRO EMS is tasked with the provision of on-scene emergency care and essential medical rescue services, and has a duty to respond to all incidents received by the emergency control centre. The Western Cape has a tiered health care model with different hospital packages of care offered at level one (district), two (regional) and three (central). The Community Perinatal Service within the Western Cape has at its core the concept of a regionalised perinatal service that ensures that all births occur within a health facility [2]. Health, social and infrastructure problems of a mixed developed and developing world result in a very high demand for EMS inter-facility transfers.

METRO EMS is assessed against a 15-min response time target for metropolitan Priority One calls and a 40-min target for rural Priority One calls [3]. Within METRO EMS, the standard of maternal and neonatal transfers has historically been reported to be poor, with clinicians expressing high levels of frustration at the prolonged response times and poorly equipped vehicles [4,5].

### The obstetric and neonatal Flying Squad

Prior to 2005, EMS provided an obstetric flying squad service that was not dedicated, but rather integrated into the general operational pool of EMS resources. However, all Flying Squad responses were logged on the control centre databases as Flying Squad calls. A more effective way to transport critical or high-risk pregnancies to a specialised unit quickly and efficiently has been needed for some time, as the previous Flying Squad Obstetric Service's satisfaction and performance ratings has deteriorated.

With the implementation of a formal METRO EMS quality assurance programme in 2005, a renewed focus on the aspects of obstetric and neonatal service was adopted. In 2006, EMS introduced a dedicated maternal and neonatal Flying Squad service to address some of these failings. The purpose of the programme was to provide service excellence in the realm of maternal and child pre-hospital care [6].

While anecdotally clinician satisfaction has dramatically increased, it is not known whether the Flying Squad service has made an impact on the way this group of patients is serviced by EMS. We therefore undertook a study to evaluate whether the introduction of a dedicated obstetric and neonatal Flying Squad transfer programme resulted in greater efficiency and improved response times within the Cape Town Metropolitan area.

## Methods

We undertook a retrospective review of all EMS obstetric and neonatal Flying Squad calls during two separate 1-year periods: 1 January - 31 December 2005 (non-dedicated Flying Squad) and 1 January - 31 December 2008 (new, dedicated Flying Squad).

### Inclusion and exclusion

All calls coded for the Flying Squad during the study period were eligible for inclusion. There were no exclusions.

### Data collection

Data are collected at the METRO EMS Cape Town Control Centre for every call received and processed in Cape Town. Data for this study were collected from the Computer Aided Dispatch programme.

### Data analysis

*Response times, transit times *and *mission times *for all maternal and neonatal transfers were examined to establish performance in the two study periods. A further comparison was made with the performance of EMS on all other Priority One calls in the same time periods (to establish whether any improvement demonstrated may have been a reflection of the general improvement achieved within METRO EMS).

Data were extracted into a password-protected Microsoft Excel (Microsoft, Redmond, WA) database from the CAD (Computer-Aided Dispatch) using a Sequel server.

Mean, median, range, standard deviation and 95% confidence intervals were used to describe different data sets. A *p*-value ≤ 0.05 was regarded as statistically significant; a chi-square test was used to compare categorical data.

A mixed models analysis was employed using SAS Systems. A repeated-measures ANOVA was used, where the year was regarded as the repeated measure and the factor was the variable.

### Measured outcomes

Definitions of terms measured are provided in the Appendix.

### Ethical considerations

Ethics approval was granted by the University of Cape Town.

## Results

### Call volume

The total number of *other P1 *calls dispatched in 2005 was 46,074; in the same period, 3,257 Flying Squad incidents were dispatched (6.6% of total P1 calls) (Table 1). In 2008, 65,885 '*other P1' *calls were dispatched (43% increase from 2005); 4,865 Flying Squad incidents were dispatched (49.3% increase, 6.9% of all P1 calls).

**Table 1:** Response time performance at 15-, 30-, 40- and 60-min intervals by case type per year

		Response count	% Response within 15	% Response within 30	% Response within 40	% Response within 60
**2005**	**Maternity/neonate**	3,257	6.1	27.8	42.8	63.2
	
	**All other P1**	46,074	15.5	50.0	64.6	80.9

**2008**	**Flying Squad**	4,865	13.2	65.1	79.0	91.6
	
	**All other P1**	65,885	24.4	67.8	80.7	92.3

### Dispatch

In 2005, 11.7% of Flying Squad calls were dispatched within 4 min and 31.9% within 10 min (Table 2). This performance is still below that recorded for all other P1 calls dispatched in both the 'under 4 min' and 'under 10 min' categories.

**Table 2:** Comparison of 2005 and 2008 dispatch times

	2005	2008	*P *value
Dispatched within 4 min	11.7%	46.6%	< 0.0001

Dispatched within 10 min	31.9%	79.3%	< 0.0001

### Response

Percentage of incidents responded to was analysed at 15, 30, 40 and 60 min (Table 1). Whilst there was a substantial improvement in the response performance achieved by the Flying Squad (*p *< 0.0001), the performance is still below that achieved by the '*all other P1' *category.

### Mean status times

Due to missing data elements for the *'time spent at hospital' *category, it was not possible to calculate the Mean Mission Time for both 2005 and 2008. Instead, 'time until arrival at the hospital' was used as a reflection of the mission time (referred to in this study as '*total pre-hospital' *time.)

In 2005, the total pre-hospital time for P1 neonatal transfers was the longest at a mean of 298 min (Table 3). This was more than twice the mean recorded for *'all other P1' *incidents in that same year. Neonatal transfers therefore spent longer in a particular status category than in any of the other categories. This trend was seen in all but one of the status categories (mean *'to hospital' *time).

**Table 3:** Mean status times as expressed in minutes by case type per year

	To dispatch (A)	To scene (B)	Response (A + B)	On scene	To hospital	Total pre-hospital
**Maternity (2005)**	32	21	53	23	22	**98**

**Maternity (2008)**	10	22	32	26	21	**79**

**Neonatal (2005)**	78	40	118	39	20	**177**

**Neonatal (2008)**	22	34	56	48	24	**128**

**Inter-facility (2005)**	58	23	81	33	20	**134**

**Inter-facility (2008)**	35	21	56	35	23	**114**

**All other calls (2005)**	22	19	41	24	15	**80**

**All other calls (2008)**	12	17	29	23	15	**67**

**Service target**	**4**	**11**	**15**	**20**	**10**	**45**

The time *'to dispatch' *was also significant with a mean of 78 min, which was nearly more than double that of P1 maternity incidents and nearly four times that recorded for '*all other' *incidents. In 2005, *'all other' *calls displayed the most efficient performance with the lowest mean time recorded in nearly all status categories. P1 maternity incidents were the next best performer with a mean '*total pre-hospital' *time of 98 min.

In 2008, performance was substantially improved across all categories, with improved mean '*pre-hospital*' times recorded for all incidents. The greatest change was observed in the P1 neonatal incidents that improved from a mean total pre-hospital time of 177 min in 2005 to 128 min in 2008. This was achieved despite a two-fold increase in call volume. The greatest performance improvement for the P1 neonatal incidents can be seen in the mean time to dispatch, which was substantially reduced from 78 min in 2005 to 22 min in 2008. However, P1 neonatal transfers still recorded the longest mean status times in most of the status categories.

P1 maternity incidents improved from a mean *'total pre-hospital' *time of 98 min in 2005 to 79 min in 2008. Significant in this improvement is the mean time recorded to dispatch the incident. This improved from 32 min in 2005 to 10 min in 2008, which was the lowest time recorded for all the categories. Inter-facility transfers recorded the worst dispatch performance with the mean time '*to dispatch' *recorded as 35 min in 2008. '*All other' *incidents recorded the best overall performance with a mean '*total pre-hospital' *time of 67 min. The reduction in time spent through the life cycle of a call is clear. This is still substantially above the service target of 45 min, indicating the continued need for improvement.

## Discussion

For the purposes of this study, improvement was defined as having completed incidents in terms of process measures, i.e., a measure of the time expended during the execution of the call. Particular focus was placed on what are regarded by METRO EMS as the most important indicators: dispatch, response and mission times. The results indicate that between 2005 and 2008, the service had made significant improvements in its performance across all incident categories as is reflected by the improvement in the '*all other P1' *categories.

The reason for this performance is likely to be found in the improved resources both in terms of staffing and of equipment. In addition, general improvements in operational structures, management capacity and personnel management may have contributed to this improvement. Improvements observed in the general ambulance operations may also reflect improvement throughout the health system as a whole. Notwithstanding, the introduction of the Flying Squad programme has resulted in significant improvements in the performance of obstetric and neonatal incidents.

### The strategy

The process adopted in the introduction of the Flying Squad model is significant for understanding its success. The engagement of key stakeholders early on, as well as their continued involvement throughout the implementation and evaluation phase, was crucial [6]. Therefore, ownership of the programme was ensured and facilitation of meeting its principle objectives would be the responsibility of all involved.

### Dispatch

It is in the analysis of the findings concerning the dispatch that the greatest impact of the Flying Squad programme is most apparent. The dramatic increase in the percentage of calls dispatched within 4 min is responsible for the bulk of the performance improvement.

Another aspect of the process that influences the success is that while it focussed on ring fencing of the ambulance and its staff, it had in fact ring fenced the dispatch of the resources as well. Efficiency of resource utilisation is built on the dispatcher's decision-making acumen, which in turn is determined by the quality and the accuracy of the information obtained. By determining the manner in which an incident is captured and evaluated (i.e. the development of predetermined criteria for dispatch), the programme has resulted in a more accurate and less vague form of communication. The result is greater dispatcher confidence and more accurate, rapid and appropriate dispatches stemming from clearly defined triage categories. This is evident in the substantial improvement in both the neonatal and maternity percentages of dispatched incidents in less than 4 min.

Perhaps one of the key questions that the study raises, and one that needs to be explored in later studies, is the question concerning dispatcher bias. The dispatcher, alone, determines which resource to activate and which P1 incident to dispatch; using his/her experience and judgement to prioritise P1 incidents before dispatching them. It is suggested that in the case of neonatal and maternity incidents, the fact that these patients are already accommodated at a health facility has led many dispatchers to defer their dispatch in favour of a primary response (such as to a road accident or patient's home).

### Response

Response time was the second process measure that was examined, and it demonstrated a significant improvement across all the incident categories evaluated. While an improvement was observed in the 15-min response performance for maternity incidents, the improvement in the neonatal category was not found to be statistically significant.

Neonatal transfers achieved a mean response time of 56 min in 2008 (118 min in 2005). The lack of available benchmarking as well as the vague definition of response times renders any meaningful comparison with times observed in other studies difficult [7]. Whilst both Kempley et al. and Abdel-Latif have reported median response times for neonatal transfers of 85 and 75 min respectively, this cannot be used as a comparison for performance achieved in this study [8,9]. Both used 'response time' as a measure from the initial discussion with the receiving facility to what they referred to as the 'first look'. They do however provide an indication of the time frames involved in executing these transfers.

Neonatal transfers have very specific requirements where safety is as important as speed of transfer. Specialised equipment is needed in terms of incubators, transport ventilators, medication and infusion pumps, etc. [10]. The Advanced Life Support (ALS) skills required to perform these transfers safely are also in high demand, further hampering a speedy execution of the transfer request. This aspect, together with the high incidence of adverse events, has meant that services need to adopt a 'stay and play' policy when dealing with these incidents. It is on this basis that the Flying Squad included in its strategy a differential response for maternity and neonatal incidents. This evolved into the use of two intermediate life support (ILS) crews to perform the maternity transfers, while the ALS crew was reserved to attend to all the neonatal and critical obstetric transfer requests.

Greater efficiency was not only seen in the dispatch and response time performance, but is also evident in the analysis of the mean status times for each of the case type categories. Most notable is the status time of the neonatal incidents in which the longest mean 'on-scene' time was observed in both 2005 and 2008. This occurred despite the significant improvement in response time performance. Kempley *et al*. and Abdel-Latif also made this observation in their analysis of retrieval teams and their performance [8,9]. This is likely due to the specialised nature of the neonatal calls. In the Flying Squad programme, this was addressed by cultivating an appropriate skill set among the crew during their 6-month rotation.

A further initiative was to ensure that each ALS crew had the necessary equipment to execute the neonatal transfers. Therefore, ambulances avoided wasting time in an attempt to locate a neonatal ventilator or working incubator. The allocation of a dedicated ambulance ensured that vehicle downtime was minimised as the crew had a greater sense of ownership and therefore took greater care. These measures ensured greater efficiency and culminated to reduce mean '*pre-hospital time'*.

### Limitations

Although the study demonstrates a significant improvement in dispatch and response times, the absence of patient outcome measures has limited the conclusions that can be made. Teams may have executed these transfers more efficiently, but the appropriateness of the dispatch or the quality of the clinical management cannot be determined. It is therefore not known whether the introduction of the Flying Squad programme provided a better level of care (which was one of the programme's key objectives).

Secondly, the Flying Squad programme does not 'stack' calls. This means that when two requests are received simultaneously, only one is allocated: a second resource is then utilised from the general ambulance operations in order to service the second call. This is part of the operating procedures for the Flying Squad, and a measure of its impact on the level of service provided is desirable. However, the frequency with which this occurs is not recorded, and therefore the impact that this has on the improvement in performance cannot be measured.

A third limitation lies in the failure to determine the time spent at the hospital during handover. In so doing, a critical component of the transfer process was ignored. Therefore, the role that the hospital has to play in enabling greater efficiency was not examined. However, during discussions at Flying Squad meetings, clinicians from referral and receiving facilities anecdotally have made no changes in standard practices when receiving these patients.

Furthermore, as this study is a retrospective analysis, the impact of potential bias on the part of the investigator cannot be ignored. More research is therefore required, examining both the process and patient outcome measures, also focussing on the establishment and validation of a morbidity and/or mortality score based on the dispatch criteria of this Flying Squad programme.

## Conclusion

The merits of a specialised retrieval team have been well established and have been met with generalised acceptance by health care systems in developed countries. The question that faces developing nations is whether or not such a programme has any role to play, taking into account the significant challenges they face in terms of struggling health care systems, poorly resourced services, significant socioeconomic burdens and poorly developed infrastructure.

The findings in this study indicate that there are significant gains to be made by developing countries through the introduction of specialist retrieval teams. The proof resides not only in the greater efficiency that it stands to benefit from, but also in understanding the operational forces that influence performance. This study has not only quantified the degree of efficiency that can be achieved, but has also highlighted several key workflow processes that are integral to performance. The evidence in support of retrieval teams is beginning to weigh in, and health care managers in developing countries need to start considering these programmes as essential components of a developing health care system.

## Conflicts of interests

The authors declare that they have no competing interests.

## Author information

**Dr. David Maritz, MBChB**: Specialist resident in Emergency Medicine, Division of Emergency Medicine, University of Cape Town and Stellenbosch University, South Africa.

**Prof. Lee Wallis, MD FRCS FCEM**: Head of the Division of Emergency Medicine, University of Cape Town and Stellenbosch University, South Africa.

**Dr. Shaheem De Vries, MBChB MPhil (EM): **METRO Emergency Medical Services

## Authors' contributions

S. De Vries came up with the study idea, collected the data, and wrote the first draft. All authors contributed to the final manuscript.

## Appendix

### Definitions of terms

Dispatch: Time 'to dispatch' is defined as the time in minutes from the receipt of the telephone call until an ambulance has been assigned to the incident. Percentage dispatches under 4 min and under 10 min were compared.

Response: 'Response' time is defined as the time in minutes from receipt of call until the vehicle arrives on scene. Response time was analysed for response under 15, 30, 40 and 60 min.

Mean status time: This is calculated using the time in minutes for each of the individual observations and then calculating the mean. This was reported for each of the different status modes: times *'to dispatch' *and *'to scene'*, as well as *'response'*, *'on-scene' *and *'to hospital' *times. No value could be determined for the time spent at the hospital due to missing data.

Inter-facility transfer: This refers to patient transfers between health care facilities and includes clinics, Community Health Centres (CHC) and hospitals.
